# Embarrassment in consumer help-seeking: how power distance belief shapes service experiences

**DOI:** 10.3389/fpsyg.2026.1690906

**Published:** 2026-07-09

**Authors:** Jixi Liang, Yao Qin

**Affiliations:** School of Business, Macau University of Science and Technology, Taipa, Macao SAR, China

**Keywords:** anticipated embarrassment, consumer help seeking, power distance belief, self-confidence, service encounter

## Abstract

**Purpose:**

Consumer help seeking occurs every day. Prior researchers have primarily examined help seeking in non-routine service encounters, such as in the education, working organization, and mental health fields, yet remain largely underexamined in daily routine consumption encounters. This research filled this gap by investigating consumers' daily help-seeking behavior from a cultural perspective, that is, how consumers' power distance belief (PDB) influences their intentions to seek assistance from service employees.

**Methods:**

We conducted three between-subject experiments with two groups of subjects from different cultural background (Asia and the United States) and three types of distinct help-seeking scenarios in daily service encounters to verify our conceptual model. ANOVA and PROCESS macro employing the bootstrapping method were used to analyze the data.

**Results:**

In study 1 we found a negative relationship between PDB and consumers' help-seeking intention using a gym equipment usage setting. In study 2 we examined the mediating role of anticipated embarrassment using a Western dinner etiquette context. In study 3 we further established the moderating role of self-confidence using an ordering wine scenario. When high-PDB consumers are high in self-confidence, they perceive weaker embarrassment, which further increases their intention to seek help from service providers compared to those with low self-confidence, and thus the negative effect of PDB on help-seeking intention decreases.

**Conclusion:**

This research helps companies better understand consumers' help-seeking behavior from a cultural perspective and enables them to more accurately design service strategies during consumer-employee interactions.

## 1 Introduction

Help seeking occurs daily across a variety of contexts. It refers to a task-oriented, conscious behavior in which individuals proactively engage in interpersonal interactions to solve problems, fulfill the needs and obtain assistance from others (Halabi et al., 2024; Lim et al., 2020). For example, diners may ask waitstaff for wine recommendations, passengers may request assistance on aircraft, and shoppers may seek help when using self-checkout machines in supermarkets. Creating a favorable consumer service delivery experience has long been identified as the primary goal for companies across the service industry due to its significant impact on consumers' future continuous engagement intentions, perceived product-service system value, consumer satisfaction and the establishment of long-term relationships with consumers (Abid et al., 2026; Cetin, 2020; Chen et al., 2020; Voorhees et al., 2017; Yam et al., 2020; Zhao and Wang, 2021). In service encounters, consumers often need to take the initiative to voice their needs so that employees can deliver timely and appropriate services (Cheung and To, 2021; Liang et al., 2020). However, difficulties or problems arise when, despite the availability of services, consumers are sometimes unwilling to express their need for help. This makes it challenging for service employees to provide the desired assistance due to an initial lack of information, which inevitably results in service failure and a poor consumption experience.

To date, prior research on consumer help seeking has largely focused on non-routine service encounters, such as those in organizational, educational, and mental health contexts (Dagani et al., 2023; Deng et al., 2024; Smalley and Hopkins, 2020; Oh et al., 2024). In contrast, consumer help seeking in everyday routine consumption settings has received limited attention. Moreover, prior help-seeking research has primarily emphasized psychological drivers, such as perceived social costs, (Halabi et al., 2024), fear of negative social evaluation (Kim et al., 2020), and threats to social image (Lannin et al., 2019; Rains et al., 2025), while largely overlooking cultural factors that may also influence consumer help-seeking. Unlike specialized help-seeking, daily routine help-seeking is more closely tied to basic social norms and common-sense rules, and involves frequent and low risk interactions. Therefore, failing to understand these commonly shared routines may make individuals feel even more incompetent, leading to a great sense of loss of control in front of public and posing a greater threat to their self-image.

Therefore, to address this gap, in the current study we will focus on help-seeking behaviors in consumers' daily routine service encounters, such as requesting help with ordering wine in restaurants, and propose that a cultural belief factor, i.e., the power distance belief (PDB), negatively affects consumers' help-seeking intention via their anticipated embarrassment.

PDB describes the extent to which individuals accept and endorse power inequalities and hierarchy in a given society (Hofstede, 2001; Winterich and Zhang, 2014). There has recently been growing research interest in the impact of PDB on consumer behavior. For example, consumers with high PDB show a decreased purchase intention for downward extended products (He et al., 2023), an increased willingness toward educational products featuring cognitive appeal (Tu et al., 2022), a greater desire for uniqueness (Qin and Wang, 2023), a stronger preference for mass-market brands (Wang et al., 2022), and a higher tendency toward conspicuous consumption (Park et al., 2023) compared to those with low PDB. In line with this research stream, we propose that consumers with high PDB are less likely to seek help from service employees compared to those with low PDB. This is because high-PDB consumers are more concerned with social status and prestige, which leads to a heightened fear of negative judgment, such as being perceived as incompetent and incapable, when requesting help from service employees.

We further propose that this effect is mediated by the anticipated embarrassment. Embarrassment is a social emotion that often stems from concerns about others' perceptions and the fear of potential negative evaluations, whether real or imagined (Krishna et al., 2019; Miller, 2001). Research suggests that embarrassment is activated when individuals experience face loss or image damage during interpersonal interactions (Jiang et al., 2023). In addition, Newman (2023) demonstrated that receiving help can cause embarrassment for the recipients. Research also pointed out that consumers are inevitably confronted with embarrassing interactions in service encounters, and may experience greater embarrassment when receiving assistance from others (Kim and Yi, 2017; Sun et al., 2023). Therefore, we propose that high-PDB consumers, who are more concerned with social status and competence, are more likely to experience anticipated embarrassment when requesting help in a service setting. As a result, they are less likely to seek help from service providers compared to those with low PDB.

Additionally, self-confidence is proposed to moderate the relationship between PDB and consumers' help-seeking intention. Individuals with high self-confidence are more optimistic, less prone to anxiety, and more willing to take risks in uncertain situations (Kao, 2024; Messaoud, 2022). In contrast, those with low self-confidence tend to be relatively pessimistic, vulnerable, risk-averse, and frequently doubt themselves (Chaouali et al., 2017; Messaoud, 2022). Therefore, when individuals have high self-confidence, even if they are high in PDB and fear embarrassment, it is likely that they are willing to interact with service providers to seek help when needed.

The current study makes several theoretical and practical contributions. First, little research on help-seeking behavior has integrated cultural hierarchy beliefs into micro psychology of daily consumer service encounter. In this paper, we propose a new cultural belief antecedent, the power distance belief, negatively influences consumers' help-seeking intention in service encounters, filling the gap both in the service marketing and cross-cultural consumer behavior literature. Second, we established a novel mediator, anticipated embarrassment, to explain the relationship between PDB and consumer help seeking. Although both PDB and embarrassment have been studied to understand their impacts on consumer behaviors, no study has examined whether PDB was positively related to embarrassment, and thus further reduced consumers' intention to seek help. Finally, self-confidence is found to be a new moderator for the above relationship, indicating that the impact of PDB on consumer help seeking would be diminished when consumers possess high self-confidence. Our study results will help service companies better understand consumers' help-seeking behaviors from a cultural perspective.

## 2 Literature review

### 2.1 Consumers' help-seeking intention

Help-seeking intention refers to a deliberate plan to make others aware of a problem or certain issue, and try to acquire corresponding assistance, support or suggestion (Halabi et al., 2024; White et al., 2018). During the past years, help-seeking has received substantial scholarly interest in organization, educational and mental health fields, as it serves a pivotal role in improving employees' job skills (Arain et al., 2020; Lin et al., 2024), enhancing students' academic performance (Smalley and Hopkins, 2020; Won et al., 2019), and alleviating people's mental health challenges (Adams et al., 2022; Song et al., 2024).

Despite these potential benefits, individuals are often reluctant to ask for help (Dagani et al., 2023; Halabi et al., 2026). Prior research identifies several psychological barriers to help-seeking, including perceived incompetence, feelings of dependence or inferiority, threats to social image, and fear of negative evaluation. For example, new hires who seek assistance from supervisors may signal incompetence or lack of independence, undermining perceptions of their competence (Deng et al., 2024). Similarly, help recipients' fear of appearing lazy and incompetent lead them to hide their difficulties, reducing their likelihood of seeking help from others (Halabi et al., 2024). In addition, fear of negative evaluation can reduce help-seeking intention. Studies indicate that seeking mental health assistance is often connected with perceived stigma, such as fears of negative social judgment, unfavorable stereotypes, and being labeled as weak or inferior, which discourages individuals to request help (Dagani et al., 2023; Kim et al., 2020). For example, Lannin et al. (2019) found that receiving mental health assistance could negatively affect individuals' self-perceptions, leading them to avoid help-seeking in order to maintain a positive self-image. Similarly, Rains et al. (2025) demonstrated that anticipated threats to face decrease help-seeking intention as individuals strive to protect their self-image. In organizational contexts, Wang and Zhang (2021) likewise found that newcomers' concern of self-image increases social anxiety, which in turn suppresses their help-seeking intentions.

Central to these barriers is the social cost associated with interaction and the potential threat to one's self-image. Because most help-seeking occurs in a social context, individuals have to publicly acknowledge a lack of capability and then initiate requests on their own (Halabi et al., 2024; Rains et al., 2025). While much is known about these professional help-seeking contexts that require expertise and knowledge, there has been limited empirical research investigating help-seeking in daily routine service encounters, especially regarding how cultural factors influence the routine help-seeking. Unlike specialized help-seeking, daily routine help-seeking is more closely tied to common-sense rules. Therefore, failing to understand these basic commonly shared routines may make individuals feel even more incompetent, leading to more severe face loss and a greater threat to their self-image.

Face concerns are closely tied to desires for social status and reputation (Su et al., 2025). Thus, help-seeking may become especially challenge for individuals who endorse power distance. Individuals high in power distance belief (PDB) are more sensitive to maintenance of authority and status, are highly motivated to avoid any behavior that lowers their social position (Hofstede, 2001; Torelli et al., 2020). As a result, we propose that PDB is negatively associated with consumers' help-seeking intentions, as the potential risk of “losing face” may suppress the benefit of help-seeking. We will elaborate it more in next section.

### 2.2 Power distance belief and consumers' help-seeking intention

PDB means individuals' belief in accepting and endorsing power inequalities and hierarchy in a given society (Hofstede, 2001). Although inequality and hierarchy exist in every society, the degree of acceptance of such inequality and hierarchy varies (Hofstede, 2001). PDB is used to measure individuals' perceptions and attitudes toward such power discrepancies (Zhang et al., 2010). Initially considered a cultural factor at the national level, PDB has more recently been studied as an individual belief variable, with a growing number of studies investigating its influences on consumer behavior. For example, consumers with a high PDB tend to purchase educational products featuring cognitive appeal, whereas those with a low PDB prefer products with emotional appeal (Tu et al., 2022). Besides, consumers high in PDB are more reluctant to buy eco-friendly fashion products (Khan et al., 2024), exhibit lower purchase intentions toward downward extended products (He et al., 2023), and favor comparable product attributes in decision-making (Lee and Cho, 2022). In addition, compared with consumers low in PDB, those high in PDB are less price-sensitive (Lee H et al., 2020), less likely to avoid in-store purchases (Lee and Lalwani, 2024), perceive lower warmth in service employees' extra-role behaviors (Wang et al., 2020), place greater emphasis on social status (Rodríguez-Bailón et al., 2020), and are more inclined to select luxury and conspicuous products (Park et al., 2023).

In the context of help seeking, requesting assistance is often stigmatized as a sign of incompetence and incapability (Lee et al., 2023). For example, research found that help seeking sometimes served as an acknowledgment of inability to solve problems independently (Halabi et al., 2024). Such an admission can further undermine an individual's desired social status and social image, and might also threaten an individual's self-efficacy, as it involves admitting inferiority to help providers (Halabi et al., 2026). Further, recent research found that competence is closely connected with social status (Louvet et al., 2024). Individuals perceived as less competent often face difficulties in acquiring resources, which in turn intensify their desire for social power and status as means of compensation (Fiske, 2018; Fiske et al., 2016; Louvet et al., 2024). Along this line, we posit that high-PDB individuals are more likely to perceive help-seeking as a threat to their social status and social image. To avoid being seen as incompetent or incapable, they may suppress their intention to seek help from service providers, prioritizing the protection of their social status over the potential benefits of assistance. Thus, we hypothesize that:

**Hypothesis 1**: Consumers high in PDB show lower help-seeking intention than those low in PDB.

### 2.3 The mediating role of anticipated embarrassment

Embarrassment, as a common social emotion, typically manifests itself in fluster, awkwardness, and shameful annoyance, stems from concerns about others' perceptions, thoughts, and the fear of possible negative judgements, whether real or imagined, leading to a decrease in self-esteem and hurt to a person's desired public image (Krishna et al., 2019; Miller, 2001). It can be invoked automatically and involuntarily due to being rooted in social interaction, and usually comes with physiological reactions such as facial flushing and neck redness (Krishna et al., 2019; Miller, 2001). Moreover, embarrassment in situations where others are present tends to threaten one's face (Jiang et al., 2023; Krishna et al., 2019).

Embarrassment differs from other negative social emotions, such as shame, guilt, and social anxiety. Shame and guilt are tied closely to severe moral wrongdoing and can be experienced in both public and private situations, while embarrassment is more often induced by the relatively trivial incidents and more occurs in public settings (Giorgetta et al., 2023; Singh and Bhushan, 2025; Tangney et al., 2007). Social anxiety is a persistent painful emotional state that is more severe than embarrassment, stemming from intense fear of negative judgment and potentially resulting in distress and depression (Miller, 2010).

In recent years, there has been a growing research interest in consumer embarrassment. Khan et al. (2023) found that purchasing conspicuous counterfeit brands could trigger consumers' embarrassment due to the fear of being recognized as counterfeit buyers, which would damage their desired image. Lee J. et al. (2020) indicated that giving small tips could prompt low-power consumers to experience anticipated embarrassment during low service quality situations because it is perceived as a sign of being cheap and poor. Additionally, consumers can experience embarrassment merely by observing others in awkward encounters (Kilian et al., 2018).

In service settings, it is increasingly likely for consumers to choose self-service or service robots, and when possible, many consumers prefer to avoid face-to-face interactions with service employees, due to the perceived embarrassment (Pitardi et al., 2021; Sun et al., 2023). Newman (2023) also demonstrated that receiving help can cause embarrassment for the recipient. Similarly, Research pointed out that consumers are inevitably confronted with embarrassing interactions in service encounters, and may experience greater embarrassment when receiving assistance from others (Kim and Yi, 2017; Sun et al., 2023). Thus, we propose that the more consumers anticipate potential embarrassment in various consumption situations, the less likely they are to seek help from service employees.

According to the PDB literature, individuals high in PDB are more sensitive to social hierarchies and are more likely to worry about violating social norms or disrespecting someone of higher status (De Mooij, 2019; Rodríguez-Bailón et al., 2020). Face theory suggests that the nature of power distance and its effects have been reflected in the notion of face, revealing the linkage between status and face (Gotzner and Mazzarella, 2021; Merkin, 2017; Su et al., 2025). Accordingly, individuals high in PDB showed a greater concern for face, including fear of losing face and the wish to enhance face, particularly in situations where they appear incompetent in front of others (Merkin, 2017; Su et al., 2025). In the context of daily service settings, there are typically frequent interactions that occur in front of employees and other consumers. This fear of losing face might amplify anticipated embarrassment in situations where individuals might make a mistake during a service encounter. For example, Ziegler et al. (2025) found that individuals with high-PDB background tend to prefer avoidance strategies when facing threatening situations, in order to prevent embarrassment and reduce loss of face. Therefore, it is reasonable to infer that endorsement of hierarchy increases sensitivity to face concerns when individuals appear unskilled in front of others, thereby amplifying embarrassment in such situations. Along this vein, we predict that consumers high in PDB will experience heightened anticipated embarrassment in situations of help-seeking, which in turn leads to a lower intention to seek help. We propose that:

**Hypothesis 2**: Anticipated embarrassment mediates the effect of PDB on consumers' help-seeking intention.

### 2.4 The moderating role of self-confidence

Self-confidence is regarded as a chronic personality trait, commonly reflecting a generalized attitude and belief toward one's capability, performance, accomplishment, and worthiness (Espinosa-Rivera et al., 2019; Oney and Oksuzoglu-Guven, 2015). Individuals high in self-confidence are found to be more optimistic, less likely to feel anxiety, and exhibiting a willingness to take risks in uncertain situations (Kao, 2024; Messaoud, 2022). Conversely, those low in self-confidence are found to be relatively pessimistic, vulnerable, risk-averse, and frequently doubt themselves (Chaouali et al., 2017; Messaoud, 2022). In addition, high self-confidence was positively related to problem solving abilities and to coping with uncertain and stressful situations (Bilgin and Ince, 2026). These characteristics align with the concept of self-efficacy, whereby individuals believe they can manage difficulties effectively (Bandura, 2023). Therefore, individuals high in self-confidence are less likely to fear social threat or experience strong embarrassment, as they trust in their ability to cope with potential challenges.

In the consumption context, a confident person is more likely to engage in interactions with service employees to meet his or her needs and demands (Jamil et al., 2021). Conversely, one with low self-confidence tends to feel less secure in his or her reactions and is more afraid of negative evaluation and rejection (Wien and Olsen, 2017). In addition, individuals' self-confidence is viewed as an important motivation for them to seek help when making uncertain and complicated decisions (Jamil et al., 2021). Furthermore, the research findings by Utkarsh et al. (2019) show that consumers with high self-confidence tend to have a stronger intention to actively search for product-related information.

**Figure 1:**
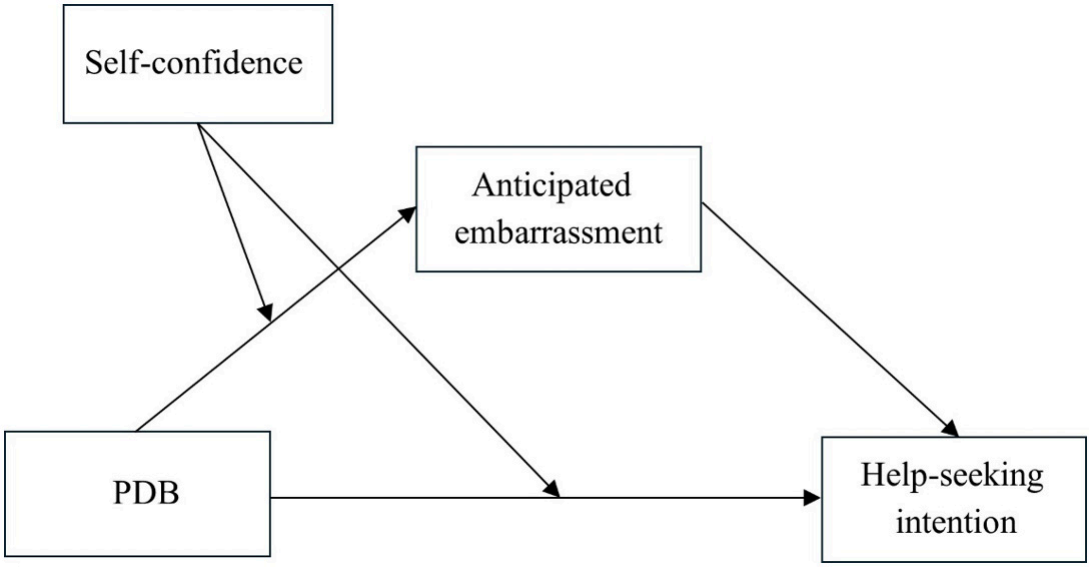
Conceptual framework.

As discussed above, PDB negatively influences consumers' help-seeking intention through anticipated embarrassment. However, this negative effect may decrease when consumers are self-confident. Self-confident consumers tend to take proactive actions to acquire information and request assistance when needed (Jamil et al., 2021; Utkarsh et al., 2019). This strong internal psychological resource can help them better manage potential social pressures in help-seeking contexts, particularly when fear of losing face and embarrassment are involved. Although high-PDB consumers typically experience heightened embarrassment, which reduces their intention to seek help, a high level of self-confidence may mitigate the perceived threat and embarrassment, making them more willing to seek help when needed. Figure 1 depicts our conceptual model. We propose that:

**Hypothesis 3**: The influence of PDB on consumers' intentions to seek help is moderated by the level of self-confidence, such that the negative effect of PDB on consumers' help-seeking intention will be attenuated through anticipated embarrassment when they have high (vs. low) self-confidence.

Next, we conducted three studies to verify the propounded theoretical model. Study 1 verified a negative relationship between PDB and help-seeking intention using a gym equipment usage setting. Study 2 explored the mediating effect of anticipated embarrassment using a Western dinner etiquette context. Study 3 further verified the boundary condition of self-confidence using a wine ordering scenario.

## 3 Study 1

The objective of study 1 was to test the relationship between PDB and consumers' help-seeking intention.

### 3.1 Participants and design

A total of 153 participants (*M*_age_ = 31.50, *SD* = 8.92; 60.10% female) were recruited from Credamo (a professional online data collection platform). We used a one factor between-subjects design (high vs. low in PDB). All of them were allocated to each of the two conditions randomly.

First, we manipulated power distance belief (PDB) following the procedure established by Song et al. (2021). Specifically, participants were presented with a campaign called “Culture Awareness Month” and encouraged to develop an understanding of diverse cultural beliefs. In the low-PDB condition, the campaign emphasized egalitarian values and guided participants to embrace equality-oriented norms, whereas in the high-PDB condition, it highlighted hierarchical values and encouraged them to appreciate hierarchy-based norms. Participants were then requested to provide reasons for supporting the hierarchical (vs. egalitarian) values and to sign their initials to strengthen the manipulation. After that, we asked them to answer three items for PDB manipulation check (α = 0.96; see Appendix; Zhang et al., 2010).

Subsequently, we manipulated consumers' help-seeking intention by asking participants to vividly imagine that they were fitness beginners, and today was their first time going to the gym for strength training. They encountered a lot of unfamiliar gym equipment with various settings and adjustments they did not know how to use. After reading above scenario, we requested them to indicate to what degree they would ask a service employee for help in operating gym equipment, employing a four-item scale adapted from Itani et al. (2020). A sample item was, “Right now, I will ask a service employee for help with how to properly use gym equipment” (1 = not at all likely, 7 = extremely likely; α = 0.93; see Appendix). We then measured their general familiarity with gym equipment using a three-items scale adapted from Olya et al. (2021) (see Appendix). A sample item was, “I often use gym equipment for training” (α = 0.95; 1 = Strongly disagree, 7 = Strongly agree). In the end, we recorded their demographic information.

### 3.2 Results

#### 3.2.1 Manipulation check

A one-way ANOVA results showed that participants primed with high-PDB perceived hierarchy as more important than those primed with low-PDB (*M*_high − PDB_ = 4.70, *SD* = 1.93, *M*_low − PDB_ = 2.07, *SD* = 1.31, *F*(1, 151) = 97.75, *p* < 0.001), showing that PDB manipulation was successful.

The results of a one sample *t*-test showed that compared to the scale midpoint, the mean value of familiarity with gym equipment was significantly low (*M* = 3.71, *SD* = 1.67; *t*(152) = −2.13, *p* < 0.05), indicating participants had the need to seek help with operating gym equipment. Thus, the design of the help-seeking scenario was effective.

#### 3.2.2 Consumers' help-seeking intention

An ANOVA results further demonstrated a significant effect of the independent variable PDB priming on dependent variable consumers' help-seeking intention (*F*(1, 151) = 22.87, *p* < 0.001). Specifically, high-PDB group was less likely to seek help from service employees (*M* = 5.41, *SD* = 1.32) than the low-PDB group (*M* = 6.18, *SD* = 0.54). H1 was thus supported. Then, a regression analysis was conducted to include demographic traits as control variables. Results indicated that the correlation between PDB and consumers' help-seeking remained significant (β = −0.34, *t*(148) = −4.46, *p* < 0.001). Age (β = 0.18, *t*(148) = 2.26, *p* < 0.05) exerted a positive impact on consumers' help-seeking intention, indicating that older consumers were more likely to ask for help from the service employees than younger consumers, the gender and income were not significant (all *F*s <1.88, *p*s > 0.06).

### 3.3 Discussion

Study 1′s results supported H1. Consumers high in PDB had a lower intention to seek help than those low in PDB.

## 4 Study 2

Study 2 sought to further replicate Study 1′s results using a new help-seeking scenario involving Western formal dinner etiquette. In addition, we will examine the mediating role of anticipated embarrassment for the above relationship.

### 4.1 Participants and design

A total of 200 participants were recruited via Credamo platform (*M*_age_ = 30.09, *SD* = 8.71; 53% female). We used a one factor (high vs. low in PDB) between-subjects design, and all participants were assigned to each of the two conditions at random.

First, the same approach used in study 1 was employed to activate PDB (Song et al., 2021). The participants then responded to the same manipulation check items for PDB priming (α = 0.96; see Appendix; Zhang et al., 2010). Subsequently, consumers' help-seeking intention was manipulated by asking them to vividly imagine that they were dining at a formal Western restaurant. After being guided to their seats by service employees, they viewed a formal western table setting consisting of plates, several different forks, knives, and glasses. Meanwhile, participants were also presented with a picture of the formal dining table setting. A pre-test with 60 participants (*M*_age_ = 28.68, *SD* = 6.88; 46.7% female) recruited from Credamo was conducted to test their familiarity with Western formal dining etiquette. The same scenario was provided. We then measured their familiarity with Western formal table dining etiquette employing three-item scales adapted from Olya et al. (2021) (1 = strongly disagree, 7 = strongly agree; α = 0.93; see the Appendix). One example question was, “I consider myself experienced in Western formal dining etiquette.” One sample *t*-test results revealed that familiarity with Western formal dining etiquette did not exceed the midpoint (*M* = 3.47, *SD* = 1.54; *t*(59) = −2.69, *p* < 0.01), indicating participants were unfamiliar with Western formal dining etiquette, and thus they might have the need to seek assistance.

After seeing the scenario, we asked them the extent to which they would ask a service employee for help with Western dining etiquette using a four-item scale (1 = not at all likely, 7 = extremely likely; α = 0.94; see Appendix) adapted from Itani et al. (2020). We next measured anticipated embarrassment by adapting Davidson et al.'s (2019) five-items scale (1 = strongly disagree, 7 = strongly agree; α = 0.95; see Appendix). One example question was, “I would feel uncomfortable asking a service employee for help with table manners.” Finally, demographic information was collected.

### 4.2 Results

#### 4.2.1 Manipulation check

A one-way ANOVA demonstrated that the high-PDB group perceived social hierarchy as more important than the low-PDB group (*M*_high − PDB_ = 5.30, *SD* = 1.60, *M*_low − PDB_ = 1.97, *SD* = 1.25, *F*(1, 198) = 270.63, *p* < 0.001), suggesting that the manipulation of power distance belief was effective.

#### 4.2.2 Consumers' help-seeking intention

The ANOVA results further demonstrated a significant effect of PDB priming on consumers' help-seeking intention (*F*(1, 198) = 15.16, *p* < 0.001). Specifically, the high-PDB group was less likely to seek help from service employees (*M* = 4.85, *SD* = 1.65) than was the low-PDB group (*M* = 5.60, *SD* = 1.16). H1 was thus supported again. Next, a regression analysis including demographic variables as control variables showed that PDB remained a significant independent variable (β = −0.26, *t*(195) = −3.81, *p* < 0.001), yet the control variables were insignificant (all *F*s <0.74, *p*s > 0.39).

#### 4.2.3 The mediating role of anticipated embarrassment

Subsequently, PROCESS model 4 with a bootstrapping method was used to examine the mediation role of anticipated embarrassment (Hayes, 2017). As expected, PDB had a significant effect on consumers' help-seeking intention via anticipated embarrassment (indirect effect = −0.63, SE = 0.15, 95% [CI] = −0.9407, −0.3538). Results further revealed that PDB was positively related to anticipated embarrassment (β = 0.33, *p* < 0.001), suggesting that consumers high in PDB perceived more embarrassment than low-PDB consumers. Furthermore, the impact of anticipated embarrassment on consumers' help-seeking intention was also significant (β = −0.64, *p* < 0.001), indicating that consumers strong in embarrassment were unwilling to seek the help from service employees. H2 was thus successfully supported.

### 4.3 Discussion

Study 2′s results supported H1 and H2. Participants high in PDB were less willing to seek help than those low in PDB. In addition, anticipated embarrassment mediated the focal relationship. Specifically, participants high in PDB perceived stronger embarrassment, which in turn lowered their intention to seek help.

## 5 Study 3

Study 3 aims to examine the moderating role of self-confidence with another new help-seeking scenario involving ordering wine at the restaurant. We predicted that the negative effect of PDB on consumer help-seeking via anticipated embarrassment would be attenuated when consumers were primed with high (vs. low) self-confidence.

### 5.1 Participants and design

We collected 398 participants in the United States (*M*_age_ = 46.04, *SD* = 14.77; 64.1% female) via Prolific. A between-subjects design with 2 (high vs. low in PDB) × 2 (high vs. low in self-confidence) conditions was used. All participants were allocated to either of the four conditions at random.

The same procedure as in the previous studies was used to prime PDB. Participants then responded to the same manipulation check items for PDB (α = 0.97; Zhang et al., 2010). Subsequently, we employed a writing task (Petty et al., 2002) to manipulate self-confidence. Specifically, in the high self-confidence condition, we asked participants to write down four past situations or experiences where they had felt very confident, accomplished, successful, and very satisfied with themselves. In the low self-confidence condition, they were requested to write down four past situations or experiences where they had felt a great deal of doubt and uncertainty about themselves, and were very dissatisfied with themselves. After that, participants responded to four self-confidence manipulation check items (1 = strongly disagree, 7 = strongly agree; α = 0.96; see Appendix) adapted from Ritchie et al. (2016). One sample item was “For the time being, I feel I have many positive qualities.”

Next, following Hanks et al.'s (2017) design, we manipulated consumers' help-seeking intention by asking subjects to visualize that they were invited to a dinner at a fine dining restaurant that they have never been to before. A well-groomed server was now approaching to provide wine service. The wine list has extensive information, such as wine names, varietals, years, regions, etc. We then asked participants to indicate the degree to which they would seek help from service employees on a three-item scale (α = 0.94; see Appendix) adapted from Itani et al. (2020). One sample item was, “Right now, I will tell a service employee that I am not knowledgeable about ordering wine and ask for help.” Next, participants were asked to self-report their anticipated embarrassment by using the same items as in Study 2 (α = 0.95; see Appendix). They also rated their familiarity with ordering wine using a three-items scale (α = 0.68; 1 = strongly disagree, 7 = strongly agree; see Appendix) adapted from Olya et al. (2021). One sample item was, “I am familiar with the wine information on the menu.” In the end, we collected their demographic information.

### 5.2 Results

#### 5.2.1 Manipulation check

A MANOVA analysis (IV: PDB and self-confidence priming; DV: PDB manipulation check) demonstrated that the perceived importance of social hierarchy was higher in the high-PDB group than in the low-PDB group (*M*_high − PDB_ = 3.60, *SD* = 2.11; *M*_low − PDB_ = 2.56, *SD* = 1.74; *F*(1, 394) = 29.08, *p* < 0.001), thus the manipulation of PDB was effective. No other effects were significant (*F*s <0.66).

A MANOVA analysis (IV: PDB and self-confidence priming; DV: PDB manipulation check) showed a significant main effect of self-confidence priming. Subjects felt more confident when they were in the high (vs. low) self-confidence group (*M*_highself − confidence_ = 5.46, *SD* = 1.37; *M*_lowself − confidence_ = 4.90, *SD* = 1.68; *F*(1, 394) = 13.02, *p* < 0.001), thus the self-confidence manipulation was effective. No other effects were significant (*F*s <0.30).

A one sample *t*-test showed that the mean value of familiarity with the wine information was significantly lower than the scale midpoint (*M* = 2.7, *SD* = 1.56; *t*(397) = −16.35, *p* < 0.001), indicating participants might need to seek help with ordering wine. Thus, the design of the help-seeking scenario was effective.

#### 5.2.2 The moderating role of self-confidence

**Figure 2:**
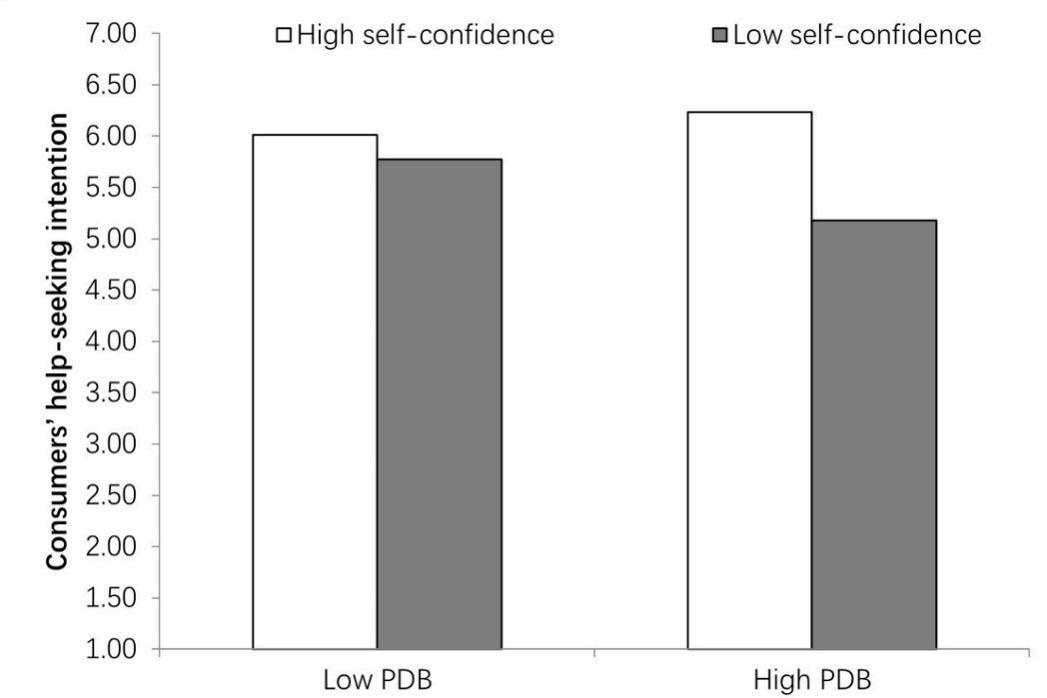
Interaction effect of PDB and self-confidence on consumers' help-seeking intention.

An ANOVA with PDB priming and self-confidence priming as the IVs and consumers' help-seeking intention as the DV exhibited a significant positive effect of self-confidence on consumers' help-seeking intention, that is, participants high in self-confidence were more willing to seek help from service employees than those low in self-confidence (*M*_highself − confidence_ = 6.11, *SD* = 1.14; *M*_lowself − confidence_ = 5.48, *SD* = 1.69; *F*(1, 394) = 20.35, *p*<*0.0*01). PDB priming did not significantly influence consumers' help-seeking intention (*M*_high − PDB_ = 5.73, *SD* = 1.59, *M*_low − PDB_ = 5.89, *SD* = 1.32, *F*(1, 394) = 1.66, *p* > 0.10). Moreover, an interaction effect of PDB priming and self-confidence priming on consumers' help-seeking intention was also significant (*F*(1, 394) = 8.1, *p* < 0.01; see Figure 2). Planned contrast results showed that participants high in PDB were more willing to seek help when they had high self-confidence than when they had low self-confidence (*M*_high − PDBhighself − confidence_ = 6.23, *M*_high − PDBlowself − confidence_ = 5.18; *F*(1, 394) = 26.77; *p* < 0.001), whereas participant low in PDB had no such difference (*p* > 0.20).

#### 5.2.3 The moderated mediation effect

Further, PROCESS Model 8 was used to conduct moderated mediation analysis (Hayes, 2017). A significant moderated mediation effect was found in bootstrapping results (index = −0.42, *SE* = 0.17, 95% CI: [−0.7730, −0.1043]). Particularly, the conditional indirect effect showed that PDB negatively influenced consumers' help-seeking intention via anticipated embarrassment, but only in low self-confidence condition (indirect effect = −0.29, *SE* =0.13, 95% CI: [−0.5617, −0.0406]); this effect was not significant in the high self-confidence condition (indirect effect = 0.13, SE = 0.1, 95% CI: [−0.0569, 0.3343]). In addition, the results also demonstrated a significant interaction effect between PDB and self-confidence on anticipated embarrassment (β = 0.57, *p* < 0.05). The conditional effect showed that PDB positively influenced anticipated embarrassment in the low self-confidence condition (effect = 0.56, SE = 0.22, 95% [CI] = 0.1281, 0.9845), but this effect was not significant in the high self-confidence condition (effect = −0.25, SE = 0.21, 95% [CI] = −0.6611, 0.1572). Moreover, the effect of anticipated embarrassment on consumers' help-seeking intention was negative and significant (β = −0.54, *p* < 0.001). H3 was thus supported.

### 5.3 Discussion

Study 3′s results supported H3. Specifically, when high-PDB consumers are high in self-confidence, they perceived weaker embarrassment, which further increased their intention to seek help from service providers compared to those with low self-confidence.

## 6 General discussion

This research extends our understanding of how cultural factors affect consumers' help-seeking intention. Three studies with three types of help-seeking encounters and two different samples (Asia and the USA) consistently demonstrated that PDB hinders consumer's help-seeking intention. Specifically, in Study 1, we established a causal relationship between PDB and consumers' help-seeking intention using a gym equipment usage scenario, that is, consumers high in PDB exhibited a lower intention to seek help than those low in PDB. This finding is consistent with previous research. Specifically, competence plays a key role in reflecting one's social status (Louvet et al., 2024), and people are reluctant to seek assistance because it signals incompetence and incapability to others (Halabi et al., 2024; Lee et al., 2023). Accordingly, hierarchy-oriented consumers show lower help-seeking intentions, as they are more attentive to status ordering and to cues that may imply low competence.

Study 2 examined the mediating effect of anticipated embarrassment using a Western formal dinner etiquette setting, that is, consumers high in PDB experienced heightened anticipated embarrassment in situations of help seeking, which in turn leads to a lower intention to seek help. The finding is consistent with prior research on embarrassment, showing that consumers are inevitably confronted with embarrassing interactions in service settings, and may experience greater embarrassment when receiving assistance from other consumers (Kim and Yi, 2017; Sun et al., 2023). In addition, individuals high in PDB showed a greater concern for face issue and social status (De Mooij, 2019; Merkin, 2017), and thus this article aligns with the Ziegler et al.'s (2025) study, suggesting that individuals with high-PDB background tend to prefer avoidance strategies when facing threatening situations, in order to prevent embarrassment and reduce loss of face.

Finally, in Study 3, we further uncovered that self-confidence moderates the above effect using ordering wine context. Specifically, when high-PDB consumers are high in self-confidence, they perceived weaker embarrassment, which further increased their intention to seek help from service providers compared to those with low self-confidence. The result aligns with the previous literature, indicating that self-confident consumers possess good coping capabilities (Bilgin and Ince, 2026), and are inclined to take proactive actions to obtain product-related information and request aid from service employees when needed (Jamil et al., 2021; Utkarsh et al., 2019). This strong internal psychological resource enables high-PDB consumers to better handle potential social pressures in help-seeking situations.

### 6.1 Theoretical contribution

First, prior research on help-seeking mainly focuses on professional and specialized service settings, such as in workplace, education, and mental clinic contexts (Dagani et al., 2023; Deng et al., 2024; Smalley and Hopkins, 2020), yet few of them examined daily routine consumption service encounters. Unlike specialized help-seeking, daily routine help-seeking is more closely anchored in basic common-sense rules, while occurring frequently and involving low risk. In addition, prior research primarily uncovered that psychological drivers, such as perceived social costs (e.g., Deng et al., 2024), fear of negative social evaluations (e.g., Kim et al., 2020), and threat of social image (e.g., Lannin et al., 2019), are the primary barriers preventing individuals to ask assistance, while largely overlooking whether and how cultural belief factors that may also influence consumer help-seeking. We in this research filled this gap by examining how power distance belief influences consumer daily routine help-seeking intention. Thus, the current study contributes to both power distance belief literature and consumers' help-seeking literature.

Secondly, we identified anticipated embarrassment as a new mediator, suggesting that consumers high (vs. low) in PDB are more likely to experience heightened embarrassment, which in turn leads to less likelihood of seeking help. Embarrassment is precisely the social emotion tied to losing face in public performance situations (Jiang et al., 2023; Miller, 2001). We identified help-seeking as a face- and status-relevant social interaction behavior. Because high PDB consumers are particularly sensitive to status and face loss, help-seeking may signal incompetence publicly, poses a threat to their face and social image. As a result, anticipated embarrassment emerges as a underlying mechanism of the relationship between PDB and help-seeking. Importantly, it differs from the other alternatives, including reduced perceived warmth of employees, decreased entitlement to request a help, and avoidance of interaction, embarrassment provides a more appropriate explanatory. First, prior research indicates that hierarchy endorsing consumers are more sensitive to competence cues rather than warmth cues (Fiske et al., 2016), and thus employees' warmth perception is more salient for low-PDB rather than high-PDB consumers (Wang et al., 2020). Second, psychological entitlement is positive related to hierarchy endorsement (Lange et al., 2018), and suggesting that status-conscious consumers may be more likely to request the help. Finally, interaction avoidance refers to generalized avoidance of communication with others in social encounters (Li et al., 2022). Yet, status-seeking consumers just selectively avoid engaging in interactions that pose a threat to their social image, while remaining willing to engage in status-enhancing interactions.

Third, self-confidence is found to be a new moderator for the above relationship, indicating that the impact of PDB on consumer help seeking would be diminished when consumers possess high self-confidence. Prior researchers found that self-confident individuals are more optimistic, more willing to engage in social interactions, more willing to take risks, and equipped with good coping skill (Bilgin and Ince, 2026; Kao, 2024; Messaoud, 2022). Along these lines, we discovered that high-PDB consumers' help-seeking intention is enhanced when they have high self-confidence than when they have low self-confidence, as they have the capacity to handle stressful situations and restore their damaged public image.

### 6.2 Managerial implications

Our research provides several important managerial implications. First, our findings help companies better understand how a cultural belief, i.e., power distance belief (PDB), impacts consumers' help-seeking intention. This understanding enables companies to design better service communication strategies during consumer-employee interactions. For example, when operating in high-PDB countries like China, Korea, India, and Japan, companies should take a more proactive approach in serving consumers instead of waiting for them to request assistance. Specifically, service providers can directly ask consumers whether they need any help.

Second, companies could reduce consumers' anticipated embarrassment to mitigate the negative relationship between PDB and help-seeking intentions, as our findings indicate that embarrassment mediates this focal relationship. For example, businesses could offer more self-service options, such as service robots and electronic tablets, instead of relying solely on human staff in service settings. When consumers need help, they can use these devices to find solutions, helping them avoid face-to-face embarrassment, particularly those with high PDB.

Finally, companies can consider using tactics to boost consumers' self-confidence, as our findings show that higher self-confidence can reduce the negative influence of PDB on the intention to ask for help. For example, businesses might create campaigns with messages like “Own Your Strength” and display them prominently. This approach can enhance consumers' self-confidence and facilitate help requests when needed.

### 6.3 Limitations and future research

Our research has certain limitations and suggestions for subsequent investigation. First, we examined only three types of consumers' help-seeking behavior. In future, researchers could explore help seeking in other service settings, such as online shopping environments. Second, in our study we examined only one boundary condition, i.e., consumers' self-confidence. In future, researchers could explore additional boundary conditions, such as social presence, to further validate the relationship between PDB and help-seeking intention. Finally, in our study we relied on three online experiments for evidence. In future, researchers might consider conducting field studies to directly observe consumers' help-seeking behavior in natural settings.

## 7 Conclusion

The current study investigated consumers' help-seeking behavior in daily service encounters from a cultural perspective, that is, how power distance belief influences their intentions to ask for assistance from service providers. Through three scenario-based experimental designs, power distance belief is found to exert a negative impact on consumers' help-seeking intention via anticipated embarrassment, and the negative effect is attenuated when consumers have high self-confidence. The research findings extended our understanding of how cultural factors affect consumers' help-seeking intention by integrating cultural hierarchy beliefs into micro psychology of daily consumer service encounter. This understanding enables companies to design better service communication strategies during consumer–employee interactions.

## Data Availability

The raw data supporting the conclusions of this article will be made available by the authors, without undue reservation.

## Appendix

**Table A1:** Constructs and measurement items.

Constructs	Items
PDB manipulation check items (Studies 1,2 and 3; Zhang et al., 2010)	1. For the time being, I am mainly thinking that... 2. At this moment, I feel that... 3. At the top of my mind right now are thoughts in agreement with saying...
Familiarity with operating gym equipment (Study1; adapted from Olya et al., 2021)	1. I often use gym equipment for training in the gym. 2. I am familiar with the operation methods of most of the gym equipment 3. I consider myself experienced in operating gym equipment.
Familiarity with Western formal dining etiquette (Study 2; adapted from Olya et al., 2021)	1. I often attend Western formal dinners. 2. I am familiar with the table manners for Western formal dining. 3. I consider myself experienced in Western formal dining etiquette.
Familiarity with ordering wine (Study 3; adapted from Olya et al., 2021)	1. I often order wine at restaurants. 2. I am familiar with the wine information on the menu. 3. I am quite knowledgeable on ordering wine.
Help-seeking intention (Studies 1 and 2; adapted from Itani et al., 2020)	1. Right now, I will ask a service employee for help with operating gym equipment (study 1)/Western dining etiquette (study 2). 2. Right now, I will communicate with a service employee to get help with operating gym equipment (study 1)/table manners (study 2). 3. Right now, I will speak with a service employee to ensure a good fitness (study 1)/dining experience (study 2). 4. Right now, I will ask a service employee for help with how to properly use gym equipment (study 1)/tableware (study 2).
Help-seeking intention (Study 3; adapted from Itani et al., 2020)	1. Right now, I will tell a service employee that I am not knowledgeable about ordering wine and ask for their help. 2. Right now, I will ask a service employee for help in choosing the appropriate wine. 3. Right now, I will ask a service employee for wine recommendations.
Anticipated embarrassment (Studies 2 and 3; adapted from Davidson et al., 2019)	1. I would feel uncomfortable asking a service employee for help with table manners (study 2)/ordering wine (study 3). 2. I would feel awkward asking a service employee for help with table manners (study 2)/ordering wine (study 3). 3. I would be concerned about being seen in public asking a service employee for help with table manners (study 2)/wine ordering (study 3). 4. I would be embarrassed if I was caught asking a service employee for help with table manners (study 2)/wine ordering (study 3). 5. I would care about what others would think if they saw me asking a service employee for help with table manners (study 2)/wine ordering (study 3).
Self-confidence manipulation check items (Study 3; adapted from Ritchie et al., 2016)	1. For the time being, I feel good about myself. 2. For the time being, I like myself better. 3. For the time being, I value myself more. 4. For the time being, I feel I have many positive qualities.
